# Nighttime Supplemental LED Inter-lighting Improves Growth and Yield of Single-Truss Tomatoes by Enhancing Photosynthesis in Both Winter and Summer

**DOI:** 10.3389/fpls.2016.00448

**Published:** 2016-04-07

**Authors:** Fasil T. Tewolde, Na Lu, Kouta Shiina, Toru Maruo, Michiko Takagaki, Toyoki Kozai, Wataru Yamori

**Affiliations:** ^1^Graduate School of Horticulture, Chiba UniversityMatsudo, Japan; ^2^Center for Environment, Health and Field Sciences, Chiba UniversityKashiwa, Japan; ^3^JA-Zen-NohKashiwa, Japan; ^4^Japan Plant Factory AssociationKashiwa, Japan; ^5^Department of Biological Sciences, Faculty of Science, The University of TokyoTokyo, Japan

**Keywords:** LED, supplemental lighting, lighting period, single-truss tomato, photosynthesis, yield, fruit quality, plant factory

## Abstract

Greenhouses with sophisticated environmental control systems, or so-called plant factories with solar light, enable growers to achieve high yields of produce with desirable qualities. In a greenhouse crop with high planting density, low photosynthetic photon flux density (PPFD) at the lower leaves tends to limit plant growth, especially in the winter when the solar altitude and PPFD at the canopy are low and day length is shorter than in summer. Therefore, providing supplemental lighting to the lower canopy can increase year-round productivity. However, supplemental lighting can be expensive. In some places, the cost of electricity is lower at night, but the effect of using supplemental light at night has not yet been examined. In this study, we examined the effects of supplemental LED inter-lighting (LED inter-lighting hereafter) during the daytime or nighttime on photosynthesis, growth, and yield of single-truss tomato plants both in winter and summer. We used LED inter-lighting modules with combined red and blue light to illuminate lower leaves right after the first anthesis. The PPFD of this light was 165 μmol m^-2^ s^-1^ measured at 10 cm from the LED module. LED inter-lighting was provided from 4:00 am to 4:00 pm for the daytime treatments and from 10:00 pm to 10:00 am for the nighttime treatments. Plants exposed only to solar light were used as controls. Daytime LED inter-lighting increased the photosynthetic capacity of middle and lower canopy leaves, which significantly increased yield by 27% in winter; however, photosynthetic capacity and yield were not significantly increased during summer. Nighttime LED inter-lighting increased photosynthetic capacity in both winter and summer, and yield increased by 24% in winter and 12% in summer. In addition, nighttime LED inter-lighting in winter significantly increased the total soluble solids and ascorbic acid content of the tomato fruits, by 20 and 25%, respectively. Use of nighttime LED inter-lighting was also more cost-effective than daytime inter-lighting. Thus, nighttime LED inter-lighting can effectively improve tomato plant growth and yield with lower energy cost compared with daytime both in summer and winter.

## Introduction

“Plant factory" refers to an environmentally controlled, plant production facility that can be divided into two types in terms of light source: plant factories with artificial light and plant factory with solar light. The former is a thermally insulated and nearly airtight warehouse-like structure in which the environment for plant growth can be controlled as precisely as desired and used for commercial production of leafy greens (e.g., lettuce etc.), herbs (e.g., perilla, mint, basil, etc.) and transplants (e.g., tomato, cucumber, paprika etc.) (Kozai, 2013; Merrill et al., 2016). The latter refers to greenhouses with sophisticated environmental control systems, or so-called plant factories with solar light, enable growers to achieve a high yield of produce with desirable qualities (Janes and McAvoy, 1991; Heuvelink et al., 2006; Yamori et al., 2014). However, predictable and consistent yields are difficult to accomplish (Cooper, 1961). In traditional multi-truss (high wire) cultivation, tomato plants have an indeterminate growth pattern and the cultural management is labor intensive (Fisher et al., 1990). Even tomato plants with 4 or 5 truss using hydroponic system combined with about 0.5 m height of benches/gutters, ladders would be needed for plant management. Single-truss tomato cultivation can drastically reduce labor requirements for training, pruning, and harvesting, and workplace ergonomics can be considerably improved by the use of high benches but demand more labor for frequent replanting and pinching (Okano et al., 2001). Moreover, single-truss tomato cultivation systems are superior to multi-truss tomato cultivation systems because they allow multiple cropping, predictable and consistent harvests, and use of moveable benches, and they have the potential for automation (Giniger et al., 1988; McAvoy and Janes, 1988; McAvoy et al., 1989; Fisher et al., 1990; Janes and McAvoy, 1991; Giacomelli et al., 1994; Logendra and Janes, 1999; Okano et al., 2001; Wada et al., 2006). Most importantly, growers using this system can capitalize on the premium paid for dependability and consistency of supply, especially for winter-produced tomatoes, which command a higher price (Janes and McAvoy, 1991).

However, light is a limiting environmental factor in the winter (Hao and Papadopoulos, 1999) and it affects photosynthesis and thus yield, since plant growth and yield depend largely on photosynthesis (Yamori, 2013; Yamori and Shikanai, 2016; Yamori et al., 2016). Because single-truss cultivation uses high plant density, light is a major limiting factor for the lower canopy leaves (Lu et al., 2012a). In winter solar light interception is limited in both top and lower canopy leaves, whereas during summer the lower canopy leaves continue to receive limited light due to the high plant density (Gunnlaugsson and Adalsteinsson, 2006), even though there is abundant light at the top of the foliar canopy (Ackerly and Bazzaz, 1995). In general, it is considered that a decrease of 1% in cumulative daily light throughout the growing season leads to a loss of 1% yield under greenhouse cultivation (Cockshull et al., 1992).

High planting density reduces light distribution along the plant profile, which is associated with mutual shading (Zhang et al., 2015). The understory leaves of tomato plants have a very low net photosynthetic rate due to both lower incident light and leaf senescence (Acock et al., 1978; Xu et al., 1997), but compared with uppermost leaves, the poor light distribution which leads to senescence had a larger effect on the photosynthetic rate of understory leaves (Acock et al., 1978). Frantz et al. (2000) suggested that supplemental light within a cowpea canopy significantly delayed senescence of the interior leaves. Supplying upward lighting from underneath also retarded the senescence of outer leaves and increased the photosynthetic rate, leading to improvement of total plant growth in lettuce (Zhang et al., 2015).

Inter-lighting can be more effective than traditional top-mounted supplemental lighting (Adams et al., 2002) and improves the net photosynthesis contribution of the lower canopy and thus yield (Hovi et al., 2004; Pettersen et al., 2010). In most cases, inter-lighting improved yield up to 50% in various crops (Hovi et al., 2004; Hovi and Tahvonen, 2008; Pettersen et al., 2010; Lu et al., 2012a,b), although some studies using different strategies and locations showed no increase in yield (Gunnlaugsson and Adalsteinsson, 2006; Heuvelink et al., 2006; Trouwborst et al., 2010). Moreover, in single-truss tomato cultivation, inter-lighting increased yield by 20% in winter and 14% in autumn (Lu et al., 2012a,b). Therefore, lighting the lower part of the canopy can be beneficial.

LEDs are considered a suitable light source for inter-lighting (Hao et al., 2012) because they produce less heat and therefore are less likely to burn leaves as compared with high-pressure sodium (HPS) lamps. In the past two decades, development of LEDs as an alternative light source has enabled not only researchers but also farmers to control spectral qualities by combining various light sources with different waveband emissions (Goto, 2003; Merrill et al., 2016). Economically efficient use of light based on the desired photosynthetic photon flux density (PPFD), lighting period (day/night cycle), and the plant growth stage when LED inter-lighting is used are key to the feasibility of the cultivation systems. The efficient plant growth stages and light qualities of inter-lighting application have been discussed (Lu et al., 2012a,b); however, no study on optimizing the lighting period with supplemental inter-lighting in winter and summer has been reported.

In addition, energy efficient use of lighting can be achieved by adjusting the LED inter-lighting schedule so that more is used at night, because the price per unit kilowatt can be lower with off-peak time-of-use (TOU), which is mostly at night. Other industries have rescheduled their operations to take advantage of discounted off-peak rates to reduce their electricity bills (Ashok and Banerjee, 2000; Ashok, 2006; Middelberg et al., 2009). In greenhouse crop production, growers are interested in alternative lighting strategies that can increase yield while reducing operating expenses. However, no study on the effect of nighttime supplemental inter-lighting on photosynthesis, growth and yield has been reported. In this study, we examined the effects of daytime and nighttime LED inter-lighting on photosynthesis, growth, and yield in a single-truss tomato cultivation system in both winter and summer.

## Materials and Methods

### Plant Material and Growth Conditions

Tomato (*Solanum lycopersicum* L., cv. Sanbi) seeds were sown in 128-cell plug trays filled with vermiculite. After 2 days in a dark room at a temperature of 26°C for germination, the trays were transferred to a walk-in type environment-controlled growth chamber (Nae Terrace, Mitsubishi Plastics Agri Dream Co., Ltd., Tokyo, Japan). Seedlings were cultivated using Otsuka nutrient solution with 2.0 ± 0.2 EC and 5.5 ∼ 6.0 pH supplied only once a day using ebb and flow system. In the growth chamber, temperature was 20/16°C day/night during the winter and 22/18°C day/night temperature in the summer, under cool white fluorescent lamps with 360 μmol m^-2^ s^-1^ PPFD and 1200 ppm CO_2_ concentration.

After 3 weeks in the growth chamber, the seedlings were transplanted in a greenhouse at a plant density of 10 plants per square meter (on 7 December 2013 and 1 August 2014 for winter and summer, respectively). The greenhouse was equipped with heat pumps to keep the day/night temperature of 22/17°C in winter, and pad and fan system were used to keep the day/night temperature at 26/20°C during the summer. The irrigation system was a modified nutrient film technique (NFT) designed to distribute nutrient solution evenly to each plant through longitudinal flow of the nutrient solution, rather than flowing from the start to the end of the growing bed (Zen-Noh Co., Ltd., Tokyo, Japan). Otsuka nutrient solution (Otsuka Chemical Co., Ltd., Osaka) was used for fertigation with an application schedule of 10 min with a subsequent 30 min break during the day and an hour break during the night. The pH was maintained at 6.0 at all times. Electrical conductivity was set at 2.5 dS m^-1^ at the time of transplanting and was increased progressively up to 12 dS m^-1^ until the harvest date in order to increase the total soluble solids content of the tomato fruits for commercial production. The tomato cultivation method was a high-plant-density single truss tomato production system in which each plant was allowed to develop only a single truss of fruit (Giacomelli et al., 1994). To achieve this, the apical meristem of each plant was pinched after the first anthesis, leaving two leaves above the first flowering truss and all flowers that set fruits were kept intact. To improve fruit setting, fully blooming flowers were sprayed once using 4-chlorophenoxy acetic acid with 15 mg L^-1^ and 7.5 mg L^-1^ concertation in summer and winter, respectively as described (Yoshida et al., 2014).

### LED Inter-lighting

**FIGURE 1:**
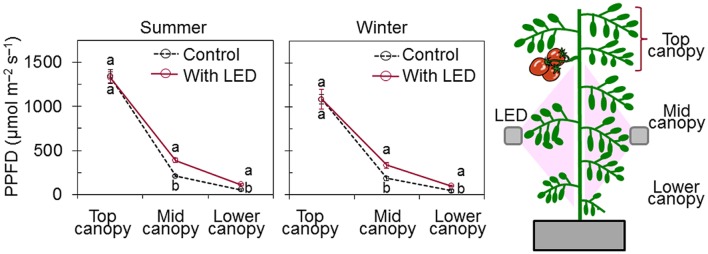
**Effect of LED inter-lighting on photosynthetic photon flux density (PPFD) along the profile of tomato plant canopy (top, middle, and lower canopy).** PPFD was measured by using a quantum sensor positioned at the inclination angle of representative canopy leaves near the point of measurement. Data represent means ± SE (*n* = 10). For each canopy level, different letters indicate statistically significant differences (*t*-test at *P* < 0.05).

LED inter-lighting (Philips Green Power LED inter-lighting module DR/B, Philips, Eindhoven, the Netherlands) was used to illuminate the understory leaves. The lamp spectrum was red and blue combined (**Supplementary Figure S1**) with a PPFD of 165 μmol m^-2^ s^-1^ measured at 10 cm from the LED module. LED modules were positioned on both sides of the aisle at a distance of 50 cm from the stem (10 cm away from the mid-canopy leaves, on average), and at a height of 40 cm from the Styrofoam panel under which the root system was established (**Figure 1**).

Previous studies reported that yield is positively correlated with the total incident light during the period from anthesis to harvest for single-truss tomato plants (McAvoy et al., 1989; Lu et al., 2012a). Therefore, we applied LED inter-lighting from the time of the very first anthesis until harvest in order to maximize yield while keeping energy cost low (from 4 September to 2 October in summer and from 16 January to 13 March in winter). To compare the electric energy use efficiency of between the daytime and nighttime LED inter-lighting schedules, we used Tokyo Electric Power Corporation’s “Otokuna night 10” as an example; this plan gives a discount rate for using power during specific off-peak periods. Plants were subjected to one of the following lighting schedules: (1) Control: solar light without LED inter-lighting, (2) daytime LED inter-lighting from 4:00 am to 4:00 pm, or (3) nighttime LED inter-lighting from 10:00 pm to 10:00 am.

### Measurements

#### Plant Growth

Internode length and stem diameter under the fruit truss, leaf chlorophyll content, leaf area, leaf mass per unit area (LMA), and shoot dry weight were measured. Chlorophyll content was determined using a spectrophotometer, as described previously (Yamori et al., 2005; Zhang et al., 2015). Leaf area was measured using an LI-3000C portable leaf area meter (Li-Cor, Lincoln, NE, USA). In addition to the growth parameters, we measured temperature and relative humidity in the microenvironment around mid-canopy by using a Thermo-hygro-CO_2_ meter (TR-76Ui; T&D Corporation, Nagano, Japan).

#### Light Distribution Along the Plant Profile

Light distribution along the plant profile was measured at each canopy level (top, middle, and lower) with a quantum sensor (LI-190SA; Li-Cor). The sensor was positioned such that the angle of inclination was the same as that of the representative canopy leaves. For LED inter-lighting measurement was made while LED inter-lighting was in use. Solar irradiance alone was used as a control and was measured similarly but in the absence of LED inter-lighting.

#### Leaf Gas Exchange

Photosynthetic rate was measured with a portable gas exchange system (LI-6400; Li-Cor) as described previously (Yamori et al., 2009, 2010). We measured the light–response curve of the photosynthetic rate, from 10:00 am to 2:00 pm under growth condition. Photosynthetic rate was measured in representative leaves from three layers of the canopy (top, middle, and lower).

Diurnal photosynthetic rate and PPFD were also measured under growth conditions using three LI-6400 gas exchange systems at the same time, one each for the control plants and plants treated with daytime and nighttime LED inter-lighting. Measurements were made using representative leaves from mid-canopy. Although the leaves were originally positioned at different distances from the LED module, the leaf cuvettes were set at a representative distance of 10 cm from the LED module. Leaves were inserted into the leaf cuvettes positioned at their original inclination angle. Diurnal PPFD was recorded by positioning quantum sensors next to each leaf subjected to diurnal photosynthetic rate measurement.

#### Yield and Fruit Quality

The fresh weight of each fruit was recorded, and two quality parameters were measured as described in Lu et al. (2015): ascorbic acid content using an RQ Flex plus (Merck Co., Ltd., Darmstadt, Germany) and total soluble solids content using a refractometer (Atago Co., Ltd., Tokyo, Japan).

#### Use of Electricity and Cost Performance

Electric energy consumption of the LED modules was measured with a multimeter and a clamp ammeter (Hioki 3169-01; Hioki E.E. Corporation, Nagano, Japan) as described previously in Zhang et al. (2015). Electric energy use efficiency was calculated as:

Electric energy use efficiency (kg kWh^-1^) = [increase in yield with LED treatment (kg m^-2^)]/[electric energy consumption (kWh m^-2^)].

Light use efficiency (g MJ^-1^) = Electric use efficiency (kg kWh^-1^)/the conversion coefficient from electrical energy to photosynthetically active radiation energy (Kozai and Niu, 2016), which is around 0.4 for recently developed LEDs (Mitchell et al., 2012).

Cost performance of LED inter-lighting was calculated as:

Cost performance (return/cost) = [price of tomato (Yen kg^-1^) × increase in yield (kg m^-2^)]/[electricity used for LED lighting per crop season (kWh m^-2^) × price of electricity (Yen kWh^-1^) + LED depreciation cost per crop season (Yen m^-2^)].

### Statistical Analysis

Data were examined using the statistical software SPSS version 21.0 (SPSS, Chicago, IL, USA); the significance of mean differences was analyzed either with Tukey’s HSD test (for more than two means) or with *t*-test (for two means).

## Results

Without LED inter-lighting, light distribution along middle and lower canopy leaves were highly deteriorated (**Figure 1**). Estimated diurnal changes in PPFD at three different canopy levels (top, middle, and lower) indicated that only 33 and 18% of the daily light reached the middle and lower foliar canopy, respectively, compared with the total incident light at the top of the foliar canopy. Introducing LED inter-lighting significantly improved the light distribution to the middle and lower canopy leaves (**Figure 1**). The light distribution within mid-canopy leaves was twice as high in plants that had direct irradiance from LED inter-lighting than in controls that were only exposed to solar light.

LED inter-lighting also altered micro-environments around the mid-canopy where there was direct irradiance. Daytime LED inter-lighting significantly increased average daytime temperature by 5.3 and 6.7% in summer and winter, respectively (**Supplementary Table S1**). Similarly, the nighttime LED inter-lighting significantly increased average nighttime temperatures, by 7.1 and 6.8% in summer and winter, respectively. In contrast, the relative humidity was significantly higher in the controls than in plants receiving nighttime LED inter-lighting treatments, in both seasons (**Supplementary Table S1**).

**FIGURE 2:**
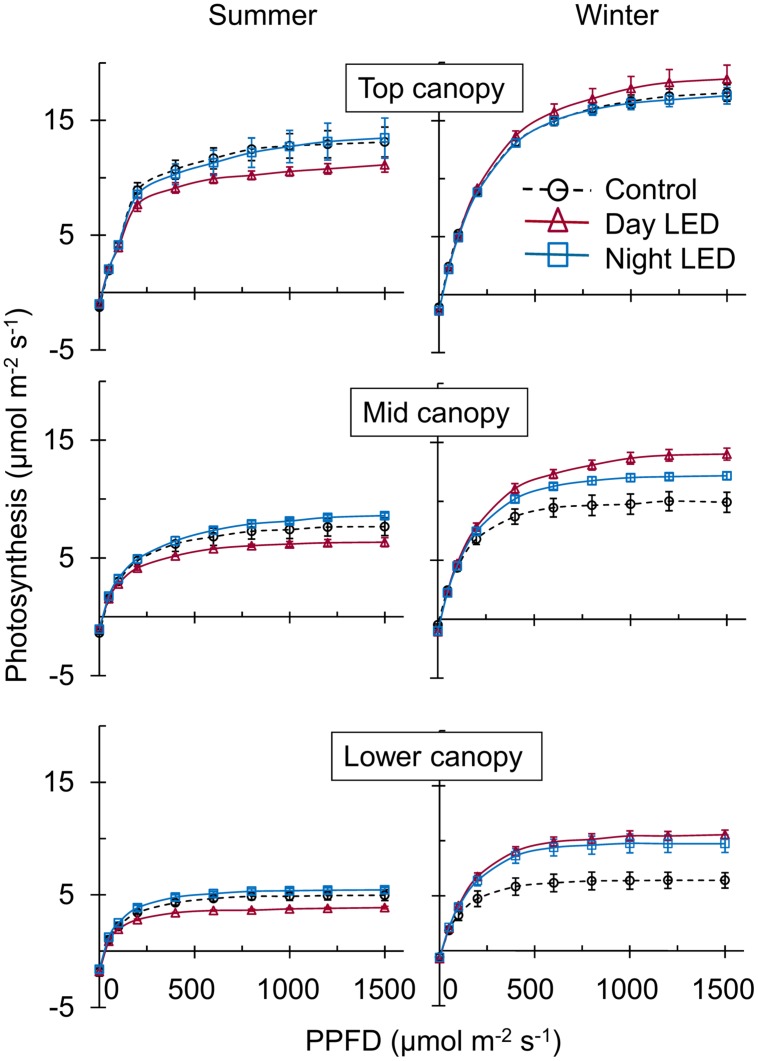
**Effect of LED inter-lighting on single-truss tomato leaf photosynthetic capacity.** Measurements were taken in representative leaves from each canopy section (top, middle, and lower) of control and LED inter-lighted plants. We measured the light–response curve of the photosynthetic rate, from 10:00 am to 2:00 pm under growth condition. Data represent means ± SE (*n* = 5).

The overall change in plant micro-environment due to nighttime LED inter-lighting was accompanied by a higher photosynthetic capacity of middle and lower canopy leaves in both summer and winter. In contrast, daytime LED inter-lighting had a positive effect on leaf photosynthetic capacity in the winter but not in the summer (**Figure 2**). The photosynthetic rates in response to growth condition PPFD were measured in mid-canopy leaves from control and both daytime and nighttime LED inter-lighting plants (**Table 1**). The mid-canopy leaf photosynthetic rate under plant growth conditions for controls at 200 μmol m^-2^ s^-1^ PPFD (without LED inter-lighting) was 4.3, and the rate for LED inter-lighting at 400 μmol m^-2^ s^-1^ PPFD was 5.2 for daytime inter-lighting and 6.4 μmol m^-2^ s^-1^ for nighttime inter-lighting (**Table 1**). The photosynthetic rate was higher in plants exposed to daytime LED inter-lighting than in controls. However, leaves treated with daytime LED inter-lighting had lower leaf photosynthetic capacity than control leaves (**Figure 2**), which offset the net photosynthetic rate under growth conditions (**Table 1**).

**Table 1:** Effect of LED inter-lighting on leaf photosynthetic rate of single-truss tomato plants.

		Mid canopy photosynthetic rate (μmol m^-2^s^-1^)		
		Light saturation	With LED	Without LED
		(1500 μmol m^-2^ s^-1^)	(400 μmol m^-2^ s^-1^)	(200 μmol m^-2^ s^-1^)
Summer	Control	7.0 ± 0.4^b^	5.6 ± 0.3^ab^	4.3 ± 0.2^a^
	Day LED	6.3 ± 0.4^b^	5.2 ± 0.1^b^	4.1 ± 0.1^a^
	Night LED	8.6 ± 0.2^a^	6.4 ± 0.3^a^	4.9 ± 0.4^a^
Winter	Control	10.0 ± 0.9^b^	8.7 ± 0.7^b^	6.8 ± 0.4^a^
	Day LED	14.0 ± 0.5^a^	11.1 ± 0.4^a^	7.8 ± 0.3^a^
	Night LED	13.1 ± 0.5^a^	10.8 ± 0.4^a^	7.8 ± 0.3^a^


*The photosynthetic rate was measured from mid-canopy leaves at 1500, 400, and 200 μmol m^-*2*^ s^-*1*^ to represent light saturation, growth conditions with LED inter-lighting, and growth conditions without LED inter-lighting, respectively, at the ambient CO_2_ concentration of 400 ppm. Data represent means ± SE (*n* = 5). Different letters indicate statistically significant differences by Tukey’s HSD (*P* < 0.05).*

**FIGURE 3:**
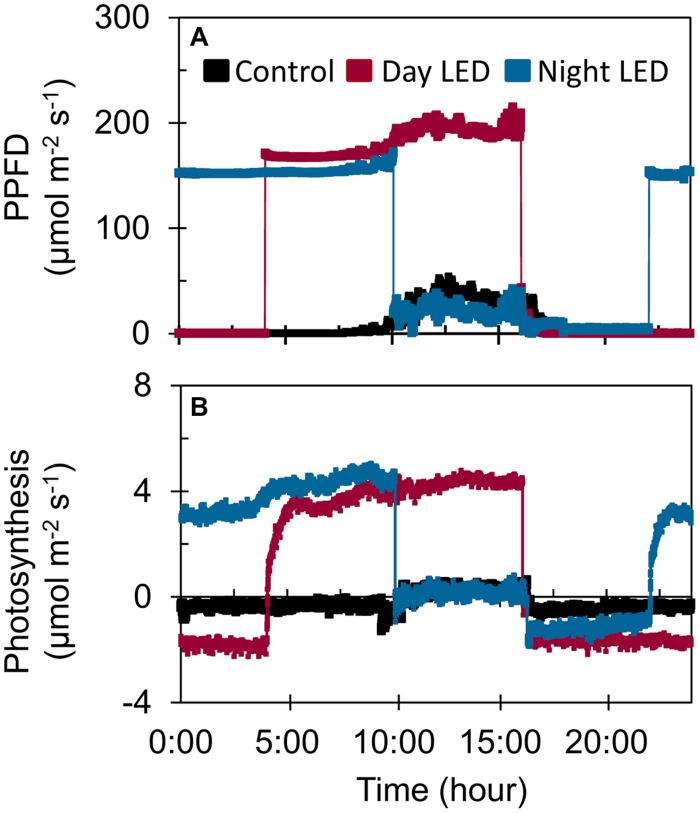
**The effect of LED inter-lighting on **(A)** diurnal photosynthetic photon flux density (PPFD) and **(B)** photosynthetic rate.** Measurements were made on 14 February using fully expanded mid-canopy leaves from control plants and plants exposed to daytime or nighttime LED inter-lighting treatments. Daytime LED inter-lighting was provided from 4:00 am to 4:00 pm and nighttime from 10:00 pm to 10:00 am.

**FIGURE 4:**
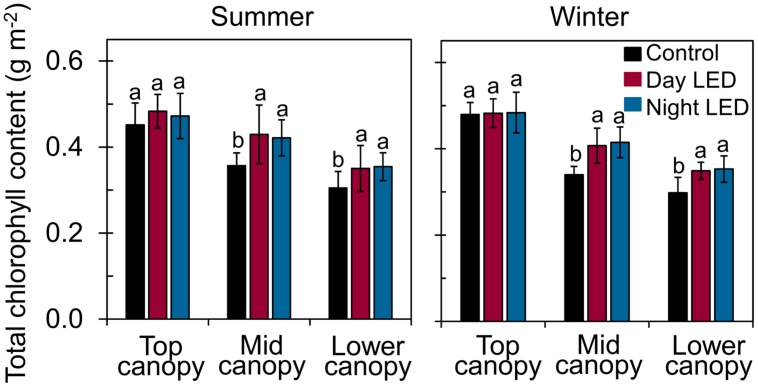
**Total chlorophyll content of single-truss tomato leaves.** Chlorophyll was measured from leaves at each level of the canopy (top, middle, and lower canopy) in plants grown under daytime or nighttime LED inter-lighting and controls, during both summer and winter. Data represent means ± SE (*n* = 15). Different letters indicate statistically significant differences (Tukey’s HSD at *P* < 0.05).

The increased light distribution along mid-canopy leaves due to daytime or nighttime LED inter-lighting (**Supplementary Figure S2**) increased the diurnal photosynthetic rate in mid-canopy leaves by 5 μmol m^-2^ s^-1^ on average, as compared with a rate of about -0.4 μmol m^-2^ s^-1^ in control plants (**Figure 3**). When both daytime and nighttime LED inter-lighting were turned off, between 4:00 pm and 10:00 pm, the leaf respiration rate was higher for leaves from plants exposed to daytime or nighttime inter-lighting than for control leaves (**Figure 3**). In addition, LED inter-lighting significantly increased chlorophyll content in both middle and lower canopy leaves, but not in top canopy leaves, (**Figure 4**), to which LED inter-lighting did not contribute PPFD (**Figure 1**). LED inter-lighting had no significant effect on leaf area index (LAI) (**Table 2**), but a significant increase in stem diameter and LMA with short internode length under the fruit truss was observed (**Table 2**).

**Table 2:** The effect of LED inter-lighting on growth of single-truss tomato plants.

		Growth parameters			
		Stem diameter (mm)	Internode length (cm)	LAI	LMA (g m^-2^)
Summer	Control	10.2 ± 0.6^b^	9.6 ± 0.7^a^	4.7 ± 0.4^a^	38.3 ± 5.3^b^
	Day LED	11.1 ± 0.9^a^	8.2 ± 0.7^b^	5.1 ± 0.5^a^	46.0 ± 1.6^a^
	Night LED	11.8 ± 0.8^a^	8.7 ± 0.9^b^	5.0 ± 0.6^a^	46.9 ± 5.3^a^
Winter	Control	11.1 ± 0.7^b^	10.6 ± 1.0^a^	3.6 ± 0.7^a^	43.1 ± 6.3^b^
	Day LED	12.2 ± 1.1^a^	9.1 ± 0.9^b^	3.7 ± 0.9^a^	47.1 ± 6.2^a^
	Night LED	11.8 ± 0.8^a^	9.3 ± 0.9^b^	3.7 ± 1.0^a^	51.5 ± 3.2^a^


*Stem diameter and internode length were measured just under the fruit truss. Both leaf area index (LAI) and leaf mass per unit area (LMA) were measured from individual leaves, and the cumulative value for a plant was used for the analysis. Data represent means ± SE (*n* = 15 for stem diameter and internode length, *n* = 5 for LAI and LMA). Different letters indicate statistically significant differences by Tukey’s HSD (*P* < 0.05).*

**FIGURE 5:**
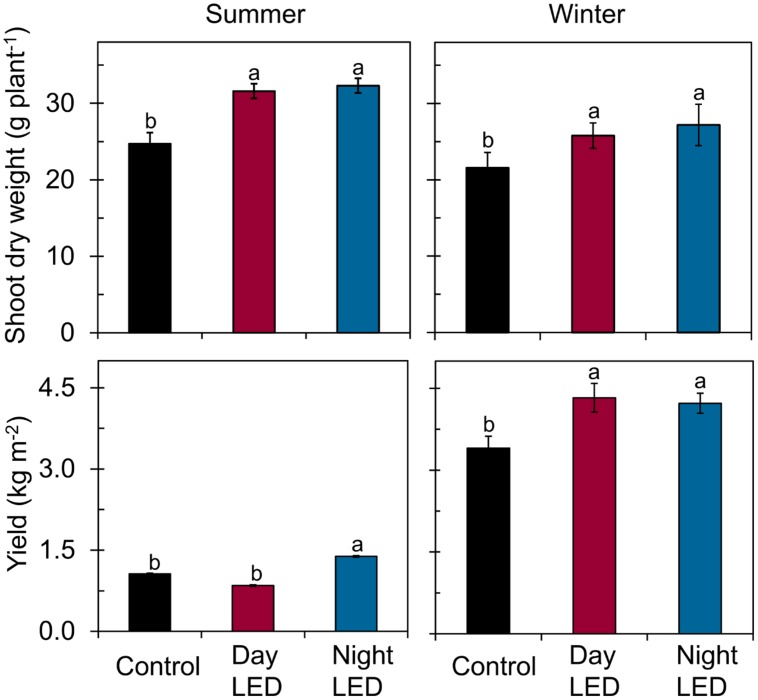
**Shoot dry weight and fruit yield of single-truss tomato plants.** Measurements were made in plants grown under daytime or nighttime LED inter-lighting or control. Data represent means ± SE (*n* = 5 and 15 for dry weight and fruit yield, respectively). Different letters indicate statistically significant differences (Tukey’s HSD at *P* < 0.05).

**FIGURE 6:**
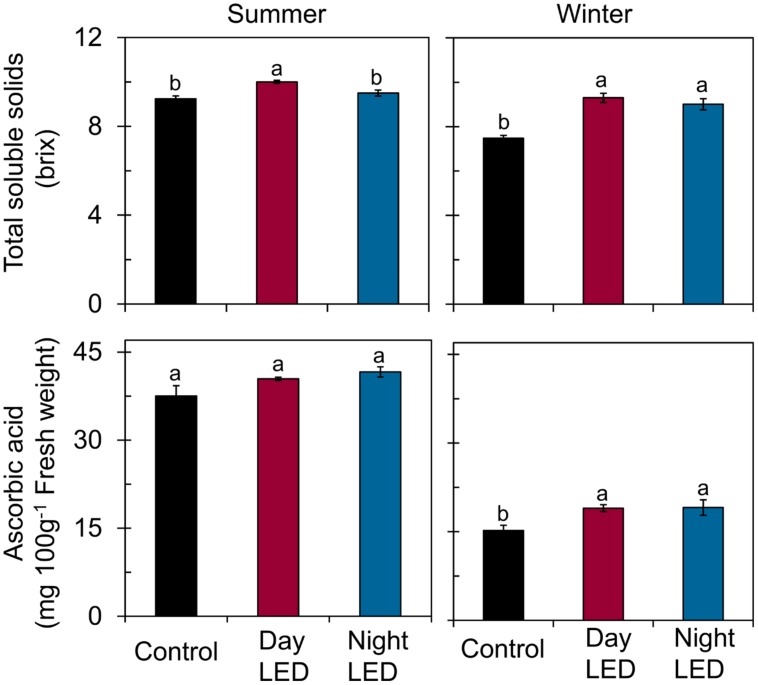
**Total soluble solids and ascorbic acid content of tomato fruits.** Measurements were made from single-truss tomato fruits grown under daytime or nighttime LED inter-lighting or control. Data represent means ± SE (*n* = 10). Different letters indicate statistically significant differences (Tukey’s HSD at *P* < 0.05).

Most importantly, we found that daytime LED inter-lighting increased yield by 27% in winter, but had no effect in summer. However, nighttime LED inter-lighting increased yield in both winter and summer by 24 and 12%, respectively (**Figure 5**). Fruit quality also significantly improved as a result of LED inter-lighting. Daytime LED inter-lighting increased total soluble solids by 8.2% in summer and 24% in winter. Nighttime LED inter-lighting significantly increased total soluble solids by 20% in winter, but the increase in summer was not significant (**Figure 6**). Winter LED inter-lighting significantly increased ascorbic acid content, by 24% for daytime inter-lighting and 25% for nighttime inter-lighting. However, neither treatment altered the ascorbic acid content during the summer (**Figure 6**).

Because we used LED inter-lighting for the same duration (12 h) for the daytime and nighttime treatments, the total electric energy used was similar, 72 kWh m^-2^ for 2 months of LED inter-lighting in winter and 36 kWh m^-2^ for 1 month of LED inter-lighting during summer. The increase in yield from LED inter-lighting during the winter was statistically equivalent for daytime (0.9 kg m^-2^) and nighttime (0.8 kg m^-2^) LED inter-lighting (**Supplementary Table S2**). In summer, nighttime LED inter-lighting increased the yield by 0.3 kg m^-2^, but daytime LED inter-lighting was deleterious, decreasing yield by 0.3 kg m^-2^ (**Supplementary Table S2**). Light use efficiency during winter for both daytime and nighttime LED lighting was 8.6 and 7.7 g MJ^-1^, respectively. In summer nighttime LED lighting had 3.9 g MJ^-1^ light use efficiency, but daytime LED light had negative effect. The cost-performance analysis for LED inter-lighting shows that only winter nighttime LED lighting can effectively improve tomato yield with high cost performance (**Supplementary Figure S3**), whereas summer nighttime and winter daytime LED lightings improved yield but the economic returns from the lighting were smaller than the running cost of electricity.

## Discussion

Development of plant factories in Japan brought single-truss tomato cultivation method for more improvement as an alternative system for its merits over multi-truss cultivation. However, at high planting density with a LAI higher than 3, light becomes a limiting factor for plant growth, because the leaf architecture and mutual shading prevent the light from penetrating to the lower canopy and thereby suppress photosynthetic activity (Okano et al., 2001). High planting density and canopy architecture plays a pivotal role for canopy light interception (Falster and Westoby, 2003; Sarlikioti et al., 2011). Therefore, providing inter-lighting to the lower leaves can be viable for year-round greenhouse crop production (Adams et al., 2002; Hovi et al., 2004; Pettersen et al., 2010). Generally, inter-lighting is based on three principles: increased light absorption in lower leaves, more efficient use of light by providing more homogeneous vertical light distribution, and preserving photosynthetic capacity of lower leaves (Giniger et al., 1988). However, it is also important to consider in which growth stage and with which lighting schedule (daytime or nighttime) inter-lighting can enhance crop yield with higher energy and cost efficiency. Previous studies showed that the yield of tomato plants increased with the increase in the total amount of PPFD received from anthesis to harvest (McAvoy et al., 1989; Lu et al., 2012a,b). However, the lighting schedule (daytime or nighttime) was not considered in previous studies.

Application of supplemental lighting when there is low light interception could improve canopy light interception, leaf chlorophyll content, and leaf photosynthetic capacity, as well as the assimilate supply to the fruit, resulting in enhanced crop yield (McAvoy and Janes, 1988; Hovi et al., 2004; Pettersen et al., 2010; Trouwborst et al., 2010). Our study clearly showed that LED inter-lighting significantly improved light distribution in the middle and lower canopy (**Figure 1**), leading to a significantly higher photosynthetic rate compared to control leaves that were grown under only solar light (**Figure 2**). This was also supported by the data that LED inter-lighting enhanced the diurnal photosynthetic rate due to better vertical light distribution (**Figure 3**). Most importantly, nighttime LED inter-lighting significantly increased tomato yield during both winter and summer, although daytime LED inter-lighting increased tomato yield only in winter (**Figure 5**). Supplemental light is expected to increase leaf photosynthesis (McAvoy and Janes, 1988; Dorias et al., 1991; Pettersen et al., 2010) and thus yield; however, in our study daytime LED inter-lighting in summer had a negative effect on leaf photosynthetic capacity which offset the net photosynthetic rate at growth condition (**Table 1**) and led to no improvement in yield (**Figure 5**). Other studies has been reported that inter-lighting did not increase yield significantly (Gunnlaugsson and Adalsteinsson, 2006; Heuvelink et al., 2006; Trouwborst et al., 2010). Trouwborst et al. (2010) explained that, the reason could be partly due to significantly reduced vertical and horizontal light interception caused by extreme leaf curling, the LED-light spectrum used, or the relatively low irradiances from above. In addition, some tomato cultivars grow equally well whether they are lit by top-lighting only or partially by inter-lighting (Gunnlaugsson and Adalsteinsson, 2006). It has also been reported that temperatures above 33°C must be avoided with most tomato cultivars when aiming to produce fruit (Adams et al., 2001; Domínguez et al., 2005). Usually supplemental lighting should be turned off when solar irradiation exceeds a desired set point, which is about 1300 μmol m^-2^ s^-1^ in a greenhouse (Dorias, 2003; Gunnlaugsson and Adalsteinsson, 2006). In our study, the average light irradiation during the middle of the day was above 1300 μmol m^-2^ s^-1^ and the temperature exceeded 33°C during the middle of the day in the summer (**Supplementary Table S1**). In addition, daytime LED inter-lighting increased light distribution and temperature significantly around mid-canopy (**Figure 1**; **Supplemental Table S1**). Therefore, it is possible that the high temperature and high solar irradiation during mid-day in summer exceeded the optimal range for tomato production and thus reduced yield.

Low light environment decreases the total soluble solids content of tomato fruit (Mc Collum, 1944; Yanagi et al., 1995). Previous studies showed that introducing inter-lighting has positive effects on fruit quality (Hovi et al., 2004), and tomato fruits exposed to high light had 35% more ascorbic acid than fruits exposed to low light (Mc Collum, 1944). In our study, daytime LED inter-lighting enhanced the total soluble solids content of single-truss tomato fruit both in winter and summer, whereas nighttime inter-lighting produced significantly higher total soluble solids than control in winter. Moreover, tomato fruit ascorbic acid content increased significantly for both daytime and nighttime inter-lighting in winter but not in summer (**Figure 6**). Among environmental factors light intensity and temperature are the most important in determining the final ascorbic acid content (Lee and Kader, 2000). In tomato, based on the promoter analysis of the genes studied by Ioannidi et al. (2009), light plays an important role in the expression of ascorbate-related genes and its gene expression was light induced with high expression during fruit development and ripening. However, summer ascorbic acid content would have been probably due to high ascorbic acid degradation in fruit at elevated temperatures with increased light exposure (Torres et al., 2006; Gautier et al., 2008).

Energy is second only to labor as the highest expense in greenhouse production (Frantz et al., 2010), and supplemental lighting may not be economically feasible (Heuvelink et al., 2006). Therefore, greenhouse crop production systems need energy-efficient lighting strategies. As a result of the increasing cost of electricity, lighting plants at night to take advantage of off-peak discounted electricity tariffs could be an alternative strategy to increase yield and decrease the cost of production. The interruption of the daily rhythm of the light-to-dark cycle might change or reset circadian rhythms in plants influencing photosynthesis (Dodd et al., 2005), but study of the canopy photosynthesis were not able to detect any disadvantage with light interrupting the night (Markvart et al., 2009). Moreover, tomatoes are day-neutral plants and photoperiod-insensitive perennials (Lifschitz and Eshed, 2006). In our study we found that nighttime LED inter-lighting increased yield and decreased energy costs in winter and summer (**Figure 5**; **Supplementary Table S2**). Nighttime LED inter-lighting had also higher light use efficiency. Previous study reported light use efficiency of tomato cultivation between 2.8 and 4.0 g MJ^-1^ (Heuvelink and Dorais, 2005). In our result, nighttime LED lighting had up to 8.6 g MJ^-1^ in winter. By taking advantage of lower off-peak electricity rates, LED inter-lighting at night could become a viable approach to increase yield and decrease cost of electricity. In our study, only winter nighttime LED lighting could be economically viable (**Supplementary Figure S3**). This suggests that whether or not to invest on supplemental LED lighting critically depends on reliable yield expectations and cost of LED for a specific condition. Fortunately, historical and projected evolution of LED is rapid and cost is decreasing; each decade, LED prices have fallen by a factor of 10 while performance has grown by a factor of 20 (Morrow, 2008).

## Conclusion

Daytime LED inter-lighting to enhance light distribution of the lower canopy leaves did not lead to higher photosynthetic capacity and yield in summer. However, photosynthesis, growth, and yield of single-truss tomatoes were improved when LED inter-lighting was provided during the daytime in winter. Nighttime LED inter-lighting had a positive effect on photosynthesis, growth, and yield in both seasons. Our results suggest that nighttime LED inter-lighting can be used cost efficiently to increase yields by taking advantage of off-peak electricity pricing, both in summer and winter. This is the first report that provides an evidence on LED inter-lighting at nighttime to take advantage of off-peak electricity discount rate.

## Author Contributions

FT, NL, and WY conceived and designed the experiments. FT performed the experiments. FT analyzed the data. FT, NL, and WY prepared the manuscript, and FT, NL, KS, TM, MT, TK, and WY contributed extensively to its finalization.

## Conflict of Interest Statement

The authors declare that the research was conducted in the absence of any commercial or financial relationships that could be construed as a potential conflict of interest.
