# Supplementary material for: Nighttime Supplemental LED Inter-lighting Improves Growth and Yield of Single-Truss Tomatoes by Enhancing Photosynthesis in Both Winter and Summer
Source: Front Plant Sci. 2016 Apr 7;7:448. doi: 10.3389/fpls.2016.00448 (PMC4823311; doi:10.3389/fpls.2016.00448)
Supplement: Supplementary file 1 [file Data_Sheet_1.PDF]

Supplementary Material:

Nighttime Supplemental LED Inter-lighting Improves Growth and Yield of Single-truss Tomatoes by Enhancing Photosynthesis in Both Winter and Summer

Fasil T. Tewolde, Na Lu, Kouta Shiina, Toru Maruo, Michiko Takagaki, Toyoki Kozai and Wataru Yamori*

*Correspondence: Wataru Yamori: wataru.yamori@bs.s.u-tokyo.ac.jp

1. SUPPLEMENTARY DATA

Supplementary Table S1-2
Supplementary Figure S1-2

2. SUPPLEMENTARY TABLES AND FIGURES

Supplementary Table 1. Effect of LED inter-lighting on temperature and humidity. Measurements were made at 5-minute intervals starting from the very first anthesis, at the same time that LED inter-lighting was initiated. Sensors were positioned within mid-canopy leaves at a distance of 20 cm from the LED module. Maximum temperature from 9:00 am to 1:00 pm was 36°C and 28°C in summer and winter, respectively. Data represent means ± SD. Different letters indicate statistically significant differences by Tukey's HSD (*P* < 0.05).

	Summer				Winter			
	Temperature (°C)		Relative humidity (%)		Temperature (°C)		Relative humidity (%)	
	4:00 am - 4:00 pm	10:00 pm -10:00 am	4:00 am - 4:00 pm	10:00 pm -10:00 am	4:00 am - 4:00 pm	10:00 pm -10:00 am	4:00 am - 4:00 pm	10:00 pm -10:00 am
Control	26.4±4.7b	21.2±3.8b	65.1±18.5a	86.1±16.2a	19.3±4.7b	14.6±2.5b	61.4±13.4a	74.0±6.0a
Day LED	27.8±4.5a	22.1±4.0ab	62.3±16.1a	84.7±16.4ab	20.6±4.9a	15.3±2.9ab	59.4±12.9a	72.5±6.5ab
Night LED	27.1±4.0ab	22.7±3.5a	64.6±14.3a	80.7±13.0b	20.1±4.4ab	15.6±2.5a	59.8±12.2a	71.1±5.9b

Supplementary Table 2. Electric energy use efficiency and light use efficiency of LED inter-lighting during summer and winter. Electric energy use efficiency = [increase in yield with LED treatment (kg m^{-2})] / [Electric energy consumption (kWh m^{-2})]. Light use efficiency = Electric use efficiency/ the conversion coefficient from electrical energy to photosynthetically active radiation energy (Kozai, 2013), which is around 0.4 for recently developed LEDs (Mitchell *et al*., 2012).

		Electric energy used (kWh m^{-2})	Yield increased (kg m^{-2})	Energy use efficiency (g kWh^{-1})	Light use efficiency (g MJ^{-1})
	Control	-	-	-	
Summer	Day LED	36.0	-0.3	-8.3	-5.7
	Night LED	36.0	0.2	5.6	3.9
	Control	-	-	-	-
Winter	Day LED	72.0	0.9	12.5	8.6
	Night LED	72.0	0.8	11.1	7.7

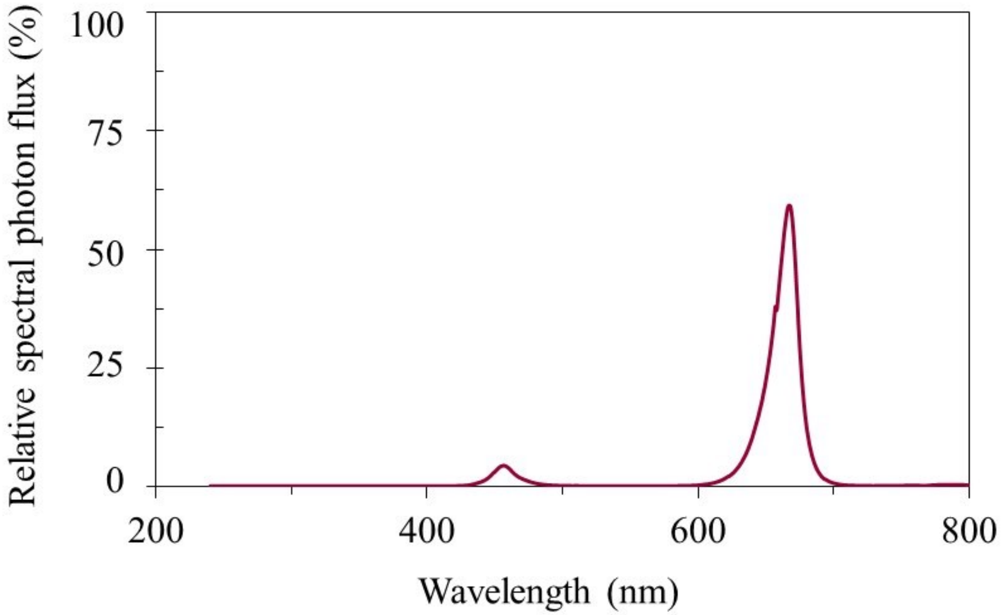

Supplementary Figure 1. The relative spectral photon flux of LED inter-lighting. Wavelength was measured between 240–800 nm with a spectroradiometer (SR9910-V7; Irradian Ltd., Tranent, UK).

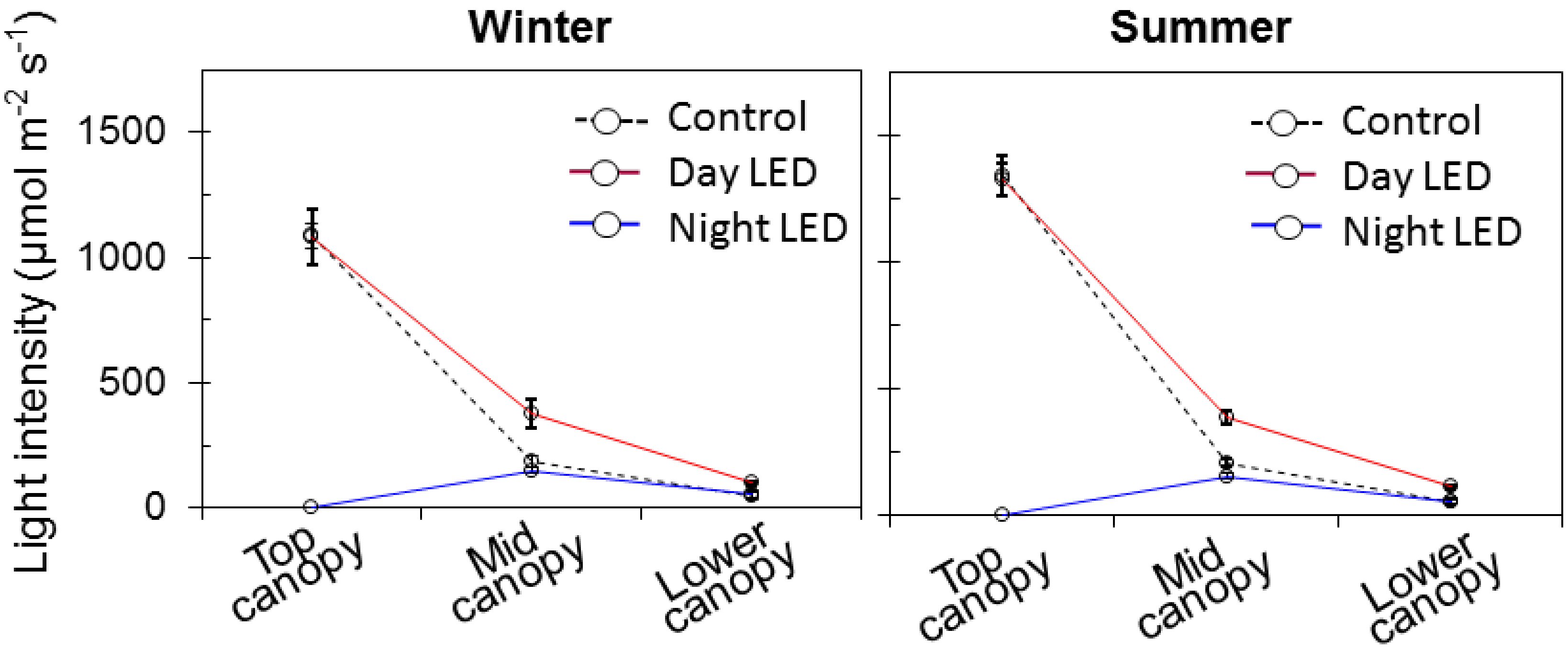

Supplementary Figure 2. Effect of LED inter-lighting on photosynthetic photon flux density (PPFD) along the profile of tomato plant canopy (top, middle, and lower canopy). PPFD was measured by using a quantum sensor positioned at the inclination angle of representative canopy leaves near the point of measurement. Data represent means ± SE (n = 10).

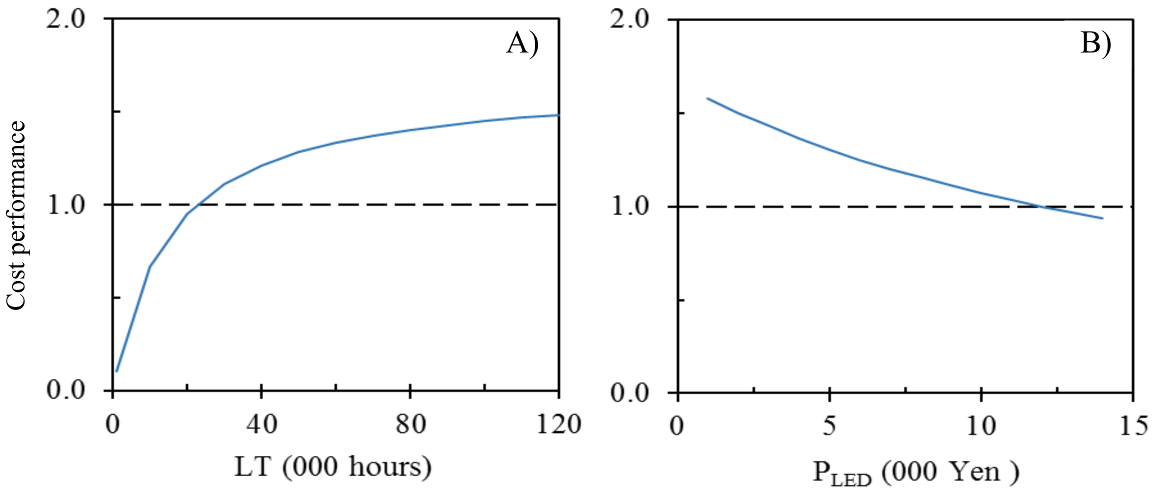

Supplementary Figure 3. Cost performance of nighttime LED inter-lighting during winter for two cases. The cost performance was defined as follows: CP = (P_y × ΔY) / (P_e × kWh + LED depreciation), where CP = cost performance (=return / cost), P_y = price of tomato (Yen kg^{-1}), ΔY = increase in tomato yield (kg m^{-2} crop $season^{-1}$), P_e = price of electricity (Yen kWh^{-1}), kWh = quantity of electricity used for LED lighting (kWh m^{-2} $crop^{-1}$), LED depreciation = initial investment cost of LED module (P_{LED}; Yen m^{-2}) / life time of LED module (in number of crops, computed as LT (life time of LED module in hours) / LED lighting hours per crop. Two cases are: Case A) that assumed the price of LED module (8000 Yen m^{-2}) with the variable life span of LED module, and Case B) that assumed the life span of LED module (50,000 hours) with the variable price of LED module.
